# Angiogenin Ameliorates Endometritis by Inhibiting NLRP3 Inflammasome Activation

**DOI:** 10.3390/ani15142002

**Published:** 2025-07-08

**Authors:** Jiangxue Cai, Yiran Sun, Hao Yang, Meiling Tan, Chenxuan Li, Lu Lu, Chenxi Liu, Bin He

**Affiliations:** 1 Key Laboratory of Animal Physiology & Biochemistry, Ministry of Agriculture and Rural Affairs, College of Veterinary Medicine, Nanjing Agricultural University, Nanjing 210095, China; caijsnow@163.com (J.C.); syr000314@163.com (Y.S.); yovmeyhz@126.com (H.Y.); 18473448821@163.com (M.T.); 2022207005@stu.njau.edu.cn (C.L.); 2023007047@stu.njau.edu.cn (L.L.); chenxiliu1111@163.com (C.L.); 2 MOE Joint International Research Laboratory of Animal Health & Food Safety, Nanjing Agricultural University, Nanjing 210095, China

**Keywords:** angiogenin, NLRP3, endometritis, inflammation

## Abstract

Endometritis adversely affects reproductive health and performance in both humans and livestock. Research demonstrates that nucleotide-binding oligomerization domain-like receptor family, pyrin domain-containing 3 (NLRP3) inflammasome activation occurs in endometritis, leading to extracellular secretion of pro-inflammatory cytokines IL-1β and IL-18 and subsequent pyroptosis. Angiogenin (Ang), a secretory vertebrate-specific ribonuclease, exhibits antimicrobial activity and has been implicated in inflammation; however, its specific role in endometritis pathogenesis remains unknown. Here, we utilized porcine endometritis models and lipopolysaccharide (LPS)-treated Ang knockout (*Ang*^−/−^) mice to investigate the function of Ang in NLRP3 inflammasome activation. The results demonstrated NLRP3 inflammasome activation in endometritis, evidenced by elevated protein levels of NLRP3, ASC, pro-Caspase-1, and cleaved Caspase-1, concurrent with Ang upregulation. Furthermore, NLRP3 inflammasome activation was significantly enhanced in LPS-treated *Ang*^−/−^ mice compared with LPS-treated wild-type (*Ang*^+/+^) controls. Mechanistically, Ang deficiency inhibited luminal epithelial cell proliferation, while recombinant Ang protein promoted proliferation in mouse endometrial epithelial cells. Collectively, these findings indicate that Ang alleviates LPS-induced endometritis by suppressing NLRP3 inflammasome activation and promotes endometrial epithelial cell proliferation during inflammation.

## Simple Summary

Endometritis, a common reproductive system disorder in swine, is primarily induced by bacterial infection and characterized by endometrial inflammation. This condition frequently results in the diminished reproductive performance of sows, adversely affecting farm economic efficiency. Although emerging evidence indicates that Angiogenin (Ang) may regulate female reproduction, its specific involvement in endometritis pathogenesis remains unexplored. This study aimed to investigate NLRP3 inflammasome activation and Ang expression dynamics during endometritis, and to elucidate the functional role of Ang in this disease via the Ang deficiency mouse model. The results demonstrate that NLRP3 inflammasome activation and Ang upregulation occur in porcine endometritis. Furthermore, Ang deficiency promoted NLRP3 inflammasome activation and exacerbated the inflammatory condition. This process appears to be mediated, at least in part, through the inhibition of endometrial epithelial cell proliferation.

## 1. Introduction

Endometritis is defined as an infection or inflammation of the endometrium, characterized by persistent inflammation of the uterine lining [1,2]. It is associated with recurrent miscarriage or repeated implantation failure. In livestock, particularly cows, mares, and sows, the increasing prevalence of endometritis has a significant impact on fertility and leads to substantial economic losses in farming operations [3]. This condition can negatively affect reproductive performance in both humans and animals. It is vital to consider more effective measures for the prevention and treatment of endometritis to improve reproductive outcomes and promote both production efficiency and animal welfare.

Previous studies have indicated that *Escherichia coli* (*E. coli*), a Gram-negative bacterium, is known to produce lipopolysaccharide (LPS), which can cause endometritis in livestock [1,2,4]. When the body is invaded by external pathogenic microorganisms, the innate immune system is activated first. Upon activation, the nucleotide-binding oligomerization domain-like receptor family, pyrin domain-containing 3 (NLRP3) inflammasome forms a multimeric protein complex, and then cleaved-Caspase-1 promotes the processing and release of mature IL-1β and IL-18 [5]. In recent years, an increasing number of studies have shown that the NLRP3 inflammasome is related to the development of female reproductive system diseases. The expression of NLRP3 has been demonstrated to influence the mesenchymal transition of endometrial epithelium, which is regulated by estrogen [6]. Furthermore, NLRP3 is highly expressed in the decidua tissue of recurrent miscarriages in women [7]. Wu et al., demonstrated that miR-495 can attenuate activation of the NLRP3 inflammasome to protect against pyroptosis and bovine endometritis [8]. Moreover, our previous study provides genetic evidence that NLRP3 ablation suppresses NLRP3 inflammasome activation in LPS-induced murine endometritis, establishing a causal role of NLRP3 in endometritis pathogenesis through reverse validation [9]. These studies suggested that the NLRP3 inflammasome plays a vital role in endometritis. A deeper understanding of the mechanism of the NLRP3 inflammasome activation will help to develop more effective treatments for inflammatory diseases.

At the cellular and molecular levels, in addition to the increase in pro-inflammatory cytokines, animals with endometritis consistently demonstrate elevated expression of antimicrobial peptides, acute phase proteins, and prostaglandins in the endometrium. Angiogenin (Ang) is a kind of antimicrobial peptide. Recent studies have indicated that Ang exhibits anti-inflammatory activity in the development and progression of inflammation [10,11,12]. Our previous study found that the expression of Ang is increased in the liver under inflammatory conditions [13]. Serum Ang protein concentration is also elevated during the acute inflammatory response [14]. A deficiency in Ang has been demonstrated to enhance susceptibility to Dextran Sulfate Sodium (DSS)-induced colitis in mice. Conversely, recombinant Ang protein protects mice from DSS-induced colitis [10]. Additionally, it can effectively inhibit the development of endotoxin-induced uveitis in a rat model by inhibiting the expression of pro-inflammatory cytokines [11]. The mechanisms by which Ang is involved in the inflammatory response may be related to the nuclear translocation of NF-κB [11,15,16]. In addition, our previous study found that Ang-mediated tsRNAs can regulate the activation of the NLRP3 inflammasome [13]. All studies have established that Ang plays a role in the pathogenesis of inflammatory diseases. However, the role of Ang in endometritis and the involved regulatory mechanisms remain unclear.

Given the vital role of Ang in inflammatory responses to bacteria, this study aimed to investigate its involvement in the inflammatory process of porcine and mouse endometritis, and whether genetic deletion of Ang influences the inflammatory reaction after LPS infection of the uterus. The findings demonstrate an unanticipated clear role for Ang in the context of endometritis and illustrate the potential for a novel therapeutic intervention in infectious endometritis.

## 2. Materials and Methods

### 2.1. Animals, Experimental Design, and Sampling

All studies were performed according to experimental protocols approved by the Institutional Animal Care and Use Committee (IACUC) of Nanjing Agricultural University. W64 French Landrace sows were kept in the farm barn at Jiangsu Huaizhou Wenshi Animal Husbandry Co., Ltd. (Huaian, China) The feeding conditions were in accordance with farm feeding requirements. Reserve sows that experienced feed reduction, repeated infertility, or uterine pus during the production process were selected as the inflammation group (Inf, n = 6). Normal reserve sows bred in the same batch were selected as the control group (Con, n = 6). Uterine tissues were examined during the slaughtering process. Uteruses with a congested appearance, tubular fluid accumulation, and pus were diagnosed with endometritis. Normal uterine tissues were collected from the Con group. The right and left uterine horns of the sows were separated and the adherent fatty tissue removed. They were then weighed separately, and the weights were recorded. The diameters of the upper, middle, and lower parts of the uterine horns were measured at fixed positions, and the average value was taken as the uterine horn diameter. The fixation samples were preserved in 4% paraformaldehyde. The molecular samples were then frozen in liquid nitrogen and stored at −80 °C for subsequent testing.

*Ang*^+/+^ C57BL/6 mice and *Ang*^−/−^ mice (Strain NO. T011609) were purchased from GemPharmatech (Nanjing, China). A previous study provides details on the precise process [13]. All mice were fed in specific pathogen-free conditions at the Laboratory Animal Center of Nanjing Agricultural University. They accessed food and water ad libitum and were housed in a 12 h light/12 h dark artificial lighting cycle room; the room temperature was 22–25 °C with 40%–60% humidity. All mice used in the experiment were 10-weeks-old. (1): female *Ang*^+/+^ C57BL/6J mice were subjected to simultaneous estrus treatment. Each mouse was injected intraperitoneally with 20 IU of pregnant mare serum gonadotropin (PMSG). Then, 48 h later, the mice were injected intraperitoneally with 5 IU of human chorionic gonadotropin (hCG) for 24 h. Endometritis was then induced by uterine instillation of 25 µL LPS (2 mg/mL, L2880, Sigma, St. Louis, MO, USA) into each uterine horn 24 h after the hCG injection (LPS). Control mice were simultaneously injected with equal amounts of PBS (CON). (2) In total, 24 female C57BL/6J mice were selected, comprising 12 *Ang*^+/+^ and 12 *Ang*^−/−^ mice. All mice were synchronized in the same way as before, in terms of their estrus cycle. After that, 6 of each gene type were randomly selected to induce endometritis with LPS. All mice were euthanized after LPS treated for 24 h, the uterine tissues were separated and the adherent fatty tissue removed. Origan index of uteri = the weight of the uterine/body weight × 100%. The uterine tissues were preserved in 4% paraformaldehyde. The molecular samples were then frozen in liquid nitrogen and stored at −80 °C for subsequent testing.

### 2.2. Isolation and Treatment of Mouse Endometrial Epithelial Cells

The luminal epithelial cells were isolated as previously described and has been improved [17]. Briefly, to obtain the luminal epithelial cells, the uteri were digested in sterile HBSS containing 9 g/L trypsin (15090046, Thermo Fisher, Waltham, MA, USA) and 6 g/L dispase (42613-33-2, Sigma, St. Louis, MO, USA) for 1 h at 4 °C, followed by a further 30 min at room temperature. After neutralization with DMEM/F12 (BR3000068, Bioleaper, Shanghai, China), the tissue block was blown through repeatedly. The cells were then gently blown into a single-cell suspension, then passed through a sterile 70 μm strainer, following centrifuge at 1000 rpm for 5 min. After washing twice with cold DMEM/F12 medium, the luminal epithelial cells were cultured in DMEM/F12 medium containing 10% heat-inactivated fetal bovine serum (FBS, Invitrogen, Waltham, MA, USA), and placed into 6-well or 96-well plates (2 × 10^5^). The purity of the endometrial epithelial cells was certified by checking cytokeratin 8 by immunohistochemistry. The purity was typically >95% (Supplementary Figure S1). For in vitro treatment, the luminal epithelial cells were treated with 10 ng/mL of recombinant Ang protein (HY-P7503, MedChemExpress, Monmouth Junction, NJ, USA) for 12 h, then treated with 500 ng/mL LPS for another 12 h.

### 2.3. Histological and Immunohistochemical Staining

The uterine horns of mice were collected after being treated with LPS for 24 h, then fixed in 4% paraformaldehyde to generate paraffin sections. For histomorphological evaluation, the sections were stained with hematoxylin/eosin (H&E). Morphological results were examined under a microscope (Ocus 40, GRUNDIUM, Helsinki, Finland). The inflammation scoring criteria are shown in Supplementary Table S1.

For immunohistochemical staining, the sections were deparaffinized in xylene and rehydrated using a graded ethanol series. The sections were incubated in a histochemistry box containing 10 mM citrate buffer (pH 6.0) at 95 °C for 15 min for antigen retrieval. Subsequently, the sections were cooled to room temperature. The sections were washed twice in distilled water. They were then incubated with 3% H_2_O_2_ at room temperature for 20 min, protected from light, and blocked with 5% BSA at 37 °C for 1 h. The sections were incubated with rabbit anti-Ki67 antibody (1:400, #12202, Cell Signaling Technology, Danvers, MA, USA) or rabbit anti-NLRP3 (1:800, #15101, Cell Signaling Technology, Danvers, MA, USA) at 4 °C overnight. Finally, the sections were incubated with the appropriate biotin-conjugated secondary antibodies, and stained with diaminobenzidine (DAB) and hematoxylin.

### 2.4. Protein Extraction and Western Blot Analysis

The uteri tissue was homogenized using 1 mL lysis buffer (P0013B, Beyotime, Shanghai, China) [containing 1× protease inhibitor (K1007, APExBIO, Houston, TX, USA) and 1× Phosphatase inhibitor (K1015, APExBIO, Houston, TX, USA)]. Then centrifuged at 4 °C, 12,000× *g* for 10 min. The protein concentration in the supernatant was measured using a BCA kit (P0012S, Beyotime, Shanghai, China). Protein samples were separated using SDS polyacrylamide gels and transferred onto polyvinylidene fluoride membrane (IPVH00010, Millipore, Burlington, MA, USA) at 100 V for 1.5 h. The membranes were then incubated with 5% milk for 2 h at room temperature. After that, membranes were incubated with the primary antibodies at 4 °C, with gentle shaking overnight. The primary antibodies used were mouse anti-Angiogenin (1:100, sc-74528, Santa, Santa cruz, CA, USA), rabbit anti-NLRP3 (ab283819, abcam, Cambridge, UK), mouse anti-Caspase1 (AG-20B-0042, AdipoGen, Epalinges, Switzerland), rabbit anti-ASC (#67824, Cell Signaling Technology, Danvers, MA, USA), and rabbit anti-Tubulinα (1:1000, AC007, ABClonal, Woburn, MA, USA). Images were captured using the VersaDoc 4000MP system (Bio-Rad, Hercules, CA, USA) and the band density was analyzed with ImageJ 1.53c.

### 2.5. Enzyme-Linked Immunosorbent Assay (ELISA)

The levels of IL-1β in mouse uterine tissue were measured using an ELISA kit (EK201B, MULTI SCIENCE, Hangzhou, China) according to the manufacturer’s instructions.

### 2.6. Myeloperoxidase (MPO) Activity Assay

The MPO activity in mouse uterine tissue was measured using MPO Detection Kit (Solarbio, Beijing, China) according to the manufacturer’s instructions.

### 2.7. EdU

To examine the effect of Ang on cell proliferation in vitro, mEECs were grown in 96-well plates as previously described. We added different concentration of recombinant Ang protein (HY-P7503, MedChemExpress, NJ, USA) in media to obtain a final concentration of 0, 10 ng/mL for 22 h. The following step used Cell Proliferation EdU Image Kit (Green Fluorescence) (KTA2030, Abbkine, Wuhan, China) as per the manufacturer’s instruction.

### 2.8. Statistical Analysis

All analyses were performed with GraphPad Prism 8.0. Data were presented as mean ± standard error of the mean (SEM). The difference between two groups was assessed using unpaired two-tailed Student’s t-test. The difference in the Ang deficiency experiment was assessed by two-way ANOVA with an uncorrected Fisher’s LSD multiple comparisons test. Statistical significance levels were denoted as follows: * *p* < 0.05, ** *p* < 0.01, *** *p* < 0.001, **** *p* < 0.0001.

## 3. Results

### 3.1. NLRP3 Inflammasome Is Activated and Ang Is Upregulated in Porcine Endometritis

**Figure 1:**
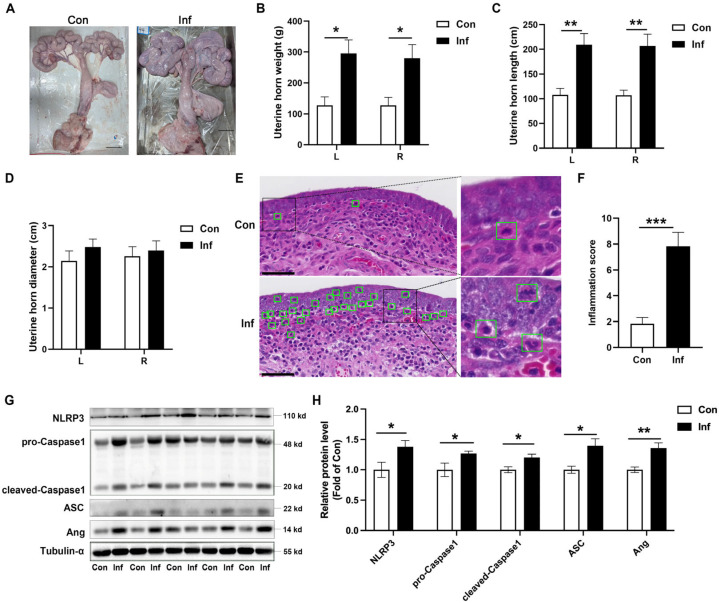
NLRP3 inflammasome is activated in porcine endometritis and Ang expression is upregulated in endometrium. (**A**) Morphological changes of the uteri. (**B**) The weight of uteri in pigs with endometritis. L: left uterine horn, R: right uterine horn. (**C**) The length of uterine tissue in pigs with endometritis. (**D**) The diameter of uterine tissue in pigs with endometritis. (**E**) Representative H&E staining sections of uteri, scale bar = 50 µm. The green box indicates the neutrophil infiltration. (**F**) Inflammation score of uteri. (**G**,**H**) Uterine tissues were analyzed by immunoblotting for NLRP3, pro-Caspase1, cleaved-Caspase1, ASC, and Ang. The values presented are mean ± SEM. The values presented are mean ± SEM, n = 6. * *p* < 0.05, ** *p* < 0.01, *** *p* < 0.001.

Pigs with endometritis exhibit a decreased appetite, anestrus or irregular estrus, infertility or miscarriage breeding, and brown mucous or purulent discharge in the uterus. The dissection revealed that the uterine tissue of sows with endometritis was enlarged, appearing congested and reddened bilaterally at the uterine horns. Inside the uterus, yellow or milky fluid was present, and the endometrium was congested (Figure 1A). The weight of the left (L) and right (R) uterine horns was significantly increased in the Inf-pig (Figure 1B), as shown by the increased length of the uterine horns (Figure 1C). However, the inflammatory process did not affect the diameter of the uterine horns (Figure 1D). There were few neutrophil infiltrations in the uterine tissue of the control group, whereas the Inf-pig showed a marked increase in neutrophil infiltration and a severe bleeding tendency (Figure 1E). Inflammation scores were higher in the uterine tissue of the Inf-pig compared to the Con-pig. (Figure 1F). Western blot analysis demonstrated a significant elevation in the expression of NLRP3, pro-Caspase1, cleaved-Caspase1, and ASC proteins in cases of porcine endometritis (Figure 1G,H and Figure S2). The expression of Ang was upregulated in pigs with endometritis (Figure 1G,H and Figure S2). All results demonstrated that NLRP3 inflammasome is activated and Ang is upregulated in porcine endometritis.

### 3.2. NLRP3 Inflammasome Is Activated and Ang Is Upregulated in LPS-Induced Murine Endometritis

**Figure 2:**
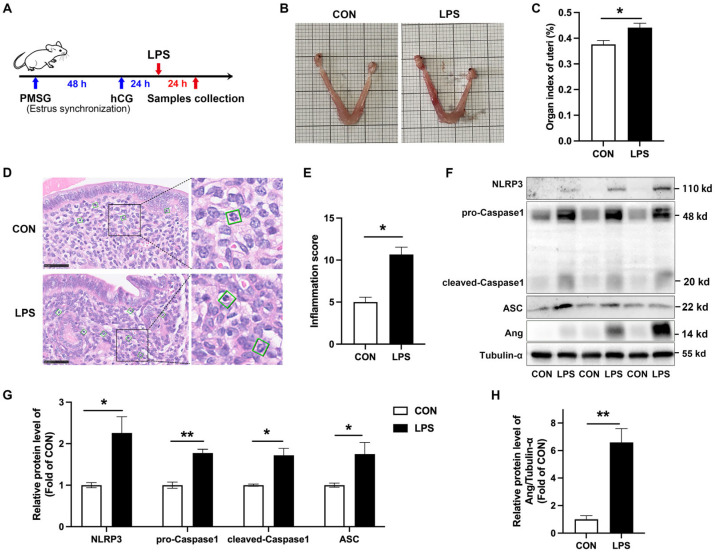
NLRP3 inflammasome is activated in mice endometritis and Ang expression is upregulated in the uteri. (**A**) Schematic diagram of the experimental design. (**B**) Morphological changes of the uteri. (**C**) Origan index of uteri. (**D**) Representative H&E staining sections of uterine tissue, scale bar = 50 µm. The green box indicates the neutrophil infiltration. (**E**) Inflammation score of uterus tissue. (**F**–**H**) The protein expression level of NLRP3, pro-Caspase1, cleaved-Caspase1, ASC, and Ang protein in the uterus. The values presented are mean ± SEM, n = 3. * *p* < 0.05, ** *p* < 0.01.

To verify this result in different animal models, LPS was injected into the uterus of mice at the site of the uterine horn to establish a model of endometritis (Figure 2A). In mice with endometritis, the uterus exhibited edema and hemorrhaging (Figure 2B). The organ index of the uterus was also increased (Figure 2C). H&E staining revealed hyperemia, hemorrhaging, and the infiltration of numerous neutrophils in the uterine tissue of LPS-treated mice (Figure 2D). Inflammation score increased significantly (Figure 2E). Western blot results showed that the NLRP3 inflammasome was activated in cases of porcine endometritis, as evidenced by an increased protein level of NLRP3, pro-Caspase1, cleaved-Caspase1, and ASC (Figure 2F,G). Ang protein levels were increased, too (Figure 2F,H). These results suggest that the NLRP3 inflammasome is activated and Ang is upregulated in mouse endometritis.

### 3.3. Ang Deficiency Aggravates LPS-Induced Murine Endometritis

**Figure 3:**
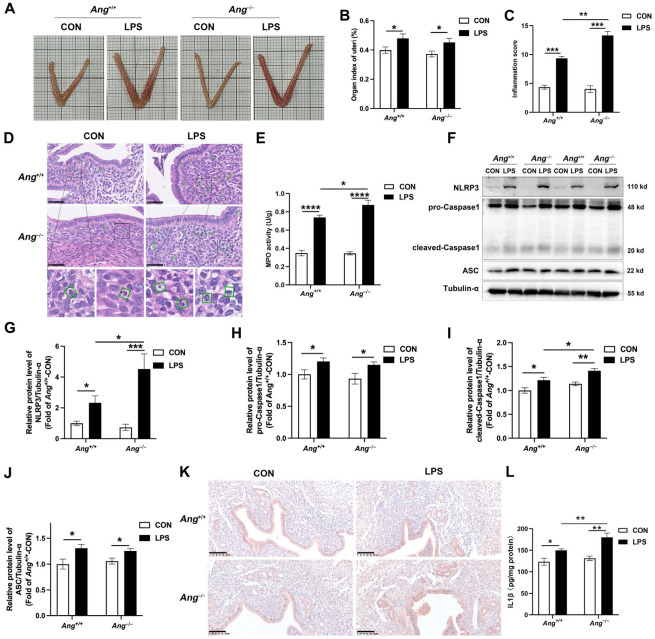
Effect of Ang knockout on LPS-induced endometritis in mice. (**A**) Morphological changes of uteri. (**B**) The organ index of uteri. (**C**) Inflammation score of uteri. (**D**) Representative H&E staining sections of uteri, scale bar = 50 µm. The green box indicates the neutrophil infiltration. (**E**) The MPO activity of uteri. (**F**–**J**) The protein expression level of NLRP3, pro-Caspase1, cleaved-Caspase1, ASC, and Ang protein in the uterus. (**K**) Immunohistochemistry of NLRP3 in the uterus. Scale bar = 100 µm. (**L**) The level of IL-1β in uterine tissue. The values presented are mean ± SEM, n = 6. * *p* < 0.05, ** *p* < 0.01, *** *p* < 0.001, **** *p* < 0.0001.

In order to investigate the effect of Ang in endometritis, both *Ang*^−/−^ and *Ang*^+/+^ C57BL/6 mice were infected with LPS for 24 h. After LPS treatment, the uterus of *Ang*^+/+^ mice showed edema and hemorrhage (Figure 3A). LPS-treated *Ang*^−/−^ mice were slightly heavier than *Ang*^+/+^ mice (Figure 3A). The uterine organ index of the LPS-treated mice was significantly higher than that of control mice, while there was no difference between the LPS-treated *Ang*^−/−^ and *Ang*^+/+^ mice (Figure 3B). The inflammation score was higher in LPS-treated mice, and that the inflammation score increased significantly in LPS-treated *Ang*^−/−^ mice compared to LPS-treated *Ang*^+/+^ mice (Figure 3C). Furthermore, treatment with LPS resulted in a significant increase in the infiltration of neutrophils. Notably, the number of neutrophils infiltrating LPS-treated *Ang*^−/−^ mice was significantly higher than in LPS-treated *Ang*^+/+^ mice (Figure 3D). MPO activity serves as a biomarker for neutrophil infiltration at the site of inflammation. LPS treatment induced a significant increase in MPO activity. The MPO activity was higher in *Ang*^−/−^ mice than in *Ang*^+/+^ mice following treatment with LPS (Figure 3E).

Consistent with porcine results, the NLRP3 inflammasome was activated in the uterine tissue of mice with endometritis, as evidenced by increased levels of the proteins NLRP3, ASC, pro-Caspase1, and cleaved-Caspase1. Compared with LPS-treated *Ang*^+/+^ mice, the protein levels of NLRP3 and cleaved-Caspase1 were higher in LPS-treated *Ang*^−/−^ mice, while the expression levels of pro-Caspase1 and ASC did not differ (Figure 3F–J and Figure S3). Furthermore, IHC analysis showed a significant increase in NLRP3 following LPS treatment, with higher NLRP3 protein levels observed in *Ang*^−/−^ mice than in *Ang*^+/+^ mice (Figure 3K). In addition, LPS treatment induced a notable increase in IL-1β levels in uterine tissues, an effect that was notably more pronounced in *Ang*^−/−^ mice (Figure 3H). Taken together, these results clearly demonstrate that the NLRP3 inflammasome is activated in the pathogenesis of endometritis, and that the uterine pathological response is enhanced in *Ang*^−/−^ mice.

### 3.4. Ang Maintains the Endometrial Health via Promoting the Proliferation of Mouse Endometrial Epithelial Cells

**Figure 4:**
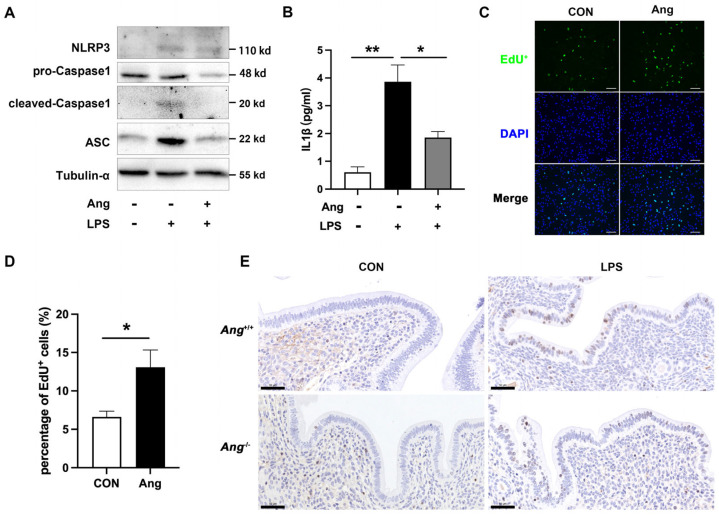
Ang alleviates endometritis via promoting the proliferation of endometrial epithelial cells. (**A**) mEECs cell extracts were analyzed by immunoblotting for NLRP3, pro-Caspase1, cleaved-Caspase1, and ASC. (**B**) Supernatants were analyzed by enzyme-linked immunosorbent assay (ELISA) for IL-1β. (**C**) Images of EdU^+^ after staining with EdU and DAPI in mEECs. Scale bars, 100 μm. (**D**) Quantification of EdU-positive cells by fluorescence image analysis. (**E**) Representative microscopic images of Ki-67 (proliferation marker) in uterine evaluated by immunohistochemistry, scale bar = 50 µm. The values presented are mean ± SEM, n = 3. * *p* < 0.05, ** *p* < 0.01.

As noted above, Ang deficiency resulted in an increase in uterine pathology and pro-inflammatory cytokine production during LPS infection. In order to explore the potential mechanism, mouse endometrial epithelial cells (mEECs) were treated with or without 10 ng/mL recombinant Ang protein and LPS. Western blot results demonstrated that recombinant Ang protein alleviates LPS-induced activation of NLRP3 inflammasome (Figure 4A). Additionally, exposure to Ang decreases the secretion of IL-1β (Figure 4B). The incorporation of EdU showed that treatment with 10 ng/mL of recombinant Ang protein for 24 h significantly enhanced cell proliferation (Figure 4C,D). Furthermore, we examined the effect of LPS infection on the expression of the proliferative marker Ki-67 in *Ang*^−/−^ and *Ang*^+/+^ mice. As shown in Figure 4E, Ki-67 expression mainly increased in the luminal epithelial cells of the uteri of LPS-treated mice. An observed decrease was also found in LPS-treated *Ang*^−/−^ mice when compared with LPS-treated *Ang*^+/+^ mice, that is, the number of Ki-67-positive cells significantly decreased in LPS-treated *Ang*^−/−^ mice. These results suggest that Ang promotes cellular proliferation in uterine luminal epithelial cells during endometritis.

## 4. Discussion

Endometritis is a common disease of the female reproductive system and is a leading cause of infertility and economic loss in the breeding industries of cattle, equines, and swine [1,18,19]. It is mainly triggered by bacterial infections, such as *Escherichia coli* and *Streptococcus*. Clinical manifestations include inflammatory reactions in the endometrium. These often lead to a decline in reproductive performance, a decrease in offspring, and an increase in the culling rates. It can also trigger systemic infections. The results observed in porcine and mouse models in this study reveal a key regulatory role of Ang in NLRP3 inflammasome activation during endometritis. In a porcine model of endometritis, NLRP3 inflammasome activation was accompanied by significantly increased Ang protein expression. This finding was replicated perfectly in the mouse model. Critically, experiments using the *Ang* knockout mouse model helped us further understand the link between Ang and the NLRP3 inflammasome: Ang deficiency leads to overactivation of the NLRP3 inflammasome. Furthermore, the proliferative state of endometrial epithelial cells progressively weakened, demonstrating that elevated Ang protein expression is a negative feedback regulatory mechanism generated by the host in response to inflammatory stress. Therefore, Ang is not only a biomarker of endometritis, but also a key endogenous inhibitory factor involved in regulating inflammatory homeostasis. Targeting Ang to enhance its function or mimic its mechanism of action may provide new therapeutic strategies.

It has been widely documented that NLRP3 inflammasome activation occurs in numerous inflammatory conditions. Notably, it occurs in autoimmune diseases and inflammatory diseases [20,21]. In our previous research, we demonstrated that the NLRP3 inflammasome was activated in mice with endometritis induced by LPS, and NLRP3 deficiency significantly reduced uterine damage [9]. In this study, we observed severe damage and significant neutrophil infiltration in the uteri of pigs and mice with endometritis, where NLRP3 inflammasome activation was also present. These findings indicate that the NLRP3 inflammasome plays a crucial role in the pathogenesis of endometritis. The activation of NLRP3 inflammatory vesicles consists of two phases: priming and activation [22]. LPS plays a key role in the priming phase, and numerous studies have demonstrated that it promotes the accumulation of NLRP3 inflammasome-associated proteins [23,24]. In our study, we found that LPS treatment promotes the accumulation of NLRP3, pro-Caspase1, and ASC proteins. The expression of these proteins is also affected by Ang deficiency and recombinant Ang protein. These results suggest that the transcription process plays an important role in Ang-mediated activation of the NLRP3 inflammasome. Therefore, targeting NLRP3 inflammasome inhibition could represent a promising therapeutic strategy for treating endometritis. Previous studies have shown that miR-495 can suppress NLRP3 inflammasome activation, protecting against pyroptosis and bovine endometritis [8]. In addition, melatonin has been found to inhibit endoplasmic reticulum (ER) stress-mediated thioredoxin interacting protein (TXNIP)/NLRP3 inflammasome activation via adenosine monophosphate-activated protein kinase (AMPK) regulation in LPS-induced endometritis [25]. Additionally, our previous findings also indicated that bile acid preconditioning alleviates LPS-induced endometritis in mice by suppressing NLRP3 inflammasome activation [9]. Taken together, these studies highlight the potential for developing new therapeutic approaches to inhibit NLRP3 activity and enhance the resolution of endometritis.

Ang was initially identified as a tumor angiogenic factor and is also recognized as an antimicrobial peptide (AMP). To date, only one Ang gene has been identified in humans; mice possess five Ang genes (Ang1, Ang2, Ang4, Ang5, and Ang6), and pigs two [26]. Hooper et al., found that mouse Ang1 and human angiogenin, which are circulating proteins upregulated during inflammation, exhibit microbicidal activity against systemic bacterial and fungal pathogens [12]. Additionally, synthetic Ang has been shown to inhibit *Mycobacterium tuberculosis* [27], *B. diminuta*, *S. paucimobilis*, *Anaerostipes* sp., and *Blautia* sp. *multiplication* [28], suggesting that Ang plays a role in the body’s systemic response to infection. In this study, we found that the expression of the Ang protein was increased in pigs and mice during endometritis. Ang deficiency resulted in severe uterine damage, and the over-activation of the NLRP3 inflammasome. These results demonstrate that *Ang* deficiency significantly exacerbates the inflammatory response in endometritis. Previous research has shown that recombinant Ang significantly attenuates the severity of experimental colitis and uveitis in vivo [10,11]. Lee et al. demonstrated that Ang reduced immune inflammation by inhibiting tank-binding kinase 1 expression in human corneal fibroblast cells [15]. Taken together, these results provide evidence that Ang inhibits NLRP3 inflammasome activation and holds promise as a potential target for the prevention and treatment of endometritis.

The proliferation status of the endometrium is likely to play a role in endometrial dysfunction. A previous study demonstrated that Ki-67 expression was significantly higher in the glandular and stromal cells of the endometrium of humans with endometritis [29]. In addition, LPS administration stimulated the proliferation of EM-E6/E7 cells derived from human endometrial cells [30]. In our study, mice with endometritis exhibited a notable increase in the proliferation in the luminal epithelial cells. Conversely, Ang deficiency was found to reduce the proliferation and activation of the NLRP3 inflammasome. Recombinant Ang protein promoted the proliferation rate in the mEECs, indicating a correlation between epithelial proliferation and NLRP3 inflammasome activation. Recent studies have demonstrated that NLRP3 inflammasome activation mediates ventilator-induced lung injury, which is accompanied by cell proliferation and trans-differentiation to compensate for alveolar membrane damage. The NIMA-related kinase 7 (NEK7) protein is a potential key regulator, and studies have demonstrated that it binds to the NLRP3 protein, promoting NLRP3 inflammasome activation [31]. NEK7 can also promote cell proliferation [32]. However, the specific mechanism of action is unclear. Therefore, we hypothesized that Ang may be a critical regulator of NEK7, inhibiting its binding to NLRP3 proteins and thereby preventing inflammatory vesicle activation whilst promoting proliferative effects. However, the specific mechanisms underlying these effects require further investigation. The mechanisms regulating cell proliferation may involve the Wnt/β-catenin and Notch signaling pathways [32]. Further research is needed to evaluate the mechanisms of cell proliferation and NLRP3 inflammasome activation.

The newly identified functions of Ang in this study suggest that it likely plays a critical role in reducing inflammation. The findings indicating the anti-inflammatory effects of Ang in LPS-treated mice shed new light on the treatment of endometritis. While this study provides valuable insights into the role of Ang in regulating endometritis, it is important to recognize the limitations. With regard to the impact of Ang deficiency on endometritis, our investigation was limited to the mice and lacked in vitro cellular-level experiments to examine the mechanism of Ang protein in the activation of NLRP3 inflammasome. Further research in this area would facilitate a more profound and sophisticated understanding of Ang’s role in immune inflammation.

## 5. Conclusions

Taking the findings from pigs and mice (especially the *Ang*^−/−^ model) together, we have demonstrated that (i) NLRP3 inflammasome is activated in porcine endometritis and LPS-induced murine endometritis; (ii) *Ang* deficiency exacerbates the activation of the NLRP3 inflammasome-related endometritis; (iii) Ang promotes the proliferation of epithelial cells, which contributes to the maintenance of endometrial health. This preliminary evidence suggests that Ang could be a strong candidate for the treatment of endometritis.

## Data Availability

All relevant data are within the manuscript.

## Supplementary Materials

The following supporting information can be downloaded at: https://www.mdpi.com/article/10.3390/ani15142002/s1, Supplementary Figure S1: Representative images showing immunofluorescence staining of CK8 in the mEECs. Supplementary Figure S2: Full Western blot image of Figure 1; Supplementary Figure S3: Full Western blot image of Figure 3. Table S1: Histopathologic scoring criteria. File S1: Full Western Blot images.
