# Supplementary material for: Angiogenin Ameliorates Endometritis by Inhibiting NLRP3 Inflammasome Activation
Source: Animals (Basel). 2025 Jul 8;15(14):2002. doi: 10.3390/ani15142002 (PMC12291823; doi:10.3390/ani15142002)
Supplement: Supplementary file 1 [file animals-15-02002-s001.zip › animals-3708800-supplementary.pdf]

animals

MDPI

Supplementary Materials

Angiogenin Ameliorates Endometritis by Inhibiting NLRP3 Inflammasome Activation

Jiangxue Cai [1], Yiran Sun [1], Hao Yang [1], Meiling Tan [1], Chenxuan Li [1], Lu Lu [1], Chenxi Liu [1], Bin He [1, 2*]

*Corresponding author

E-mail: heb@njau.edu.cn

This file includes:

Supplementary Figures S1 to S3

Supplementary Table S1

File S1: Full Western Blot images

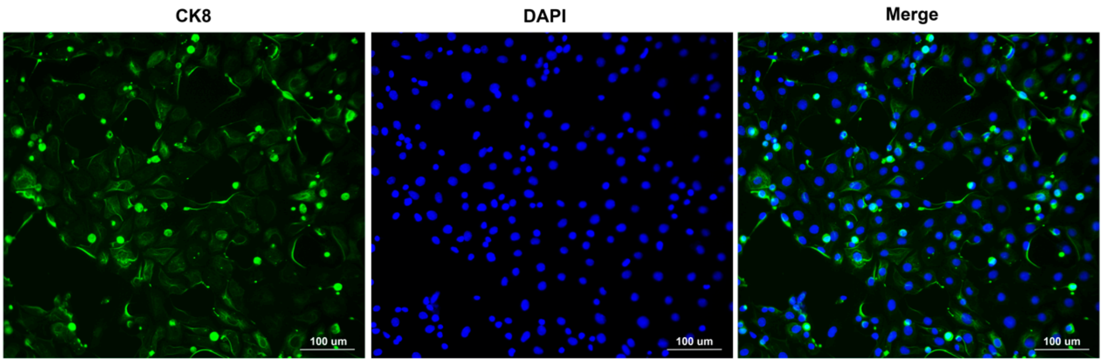

Supplementary Figure S1

Representative images showing immunofluorescence staining of CK8 in the mEECs. Scale bars, 100 μm.

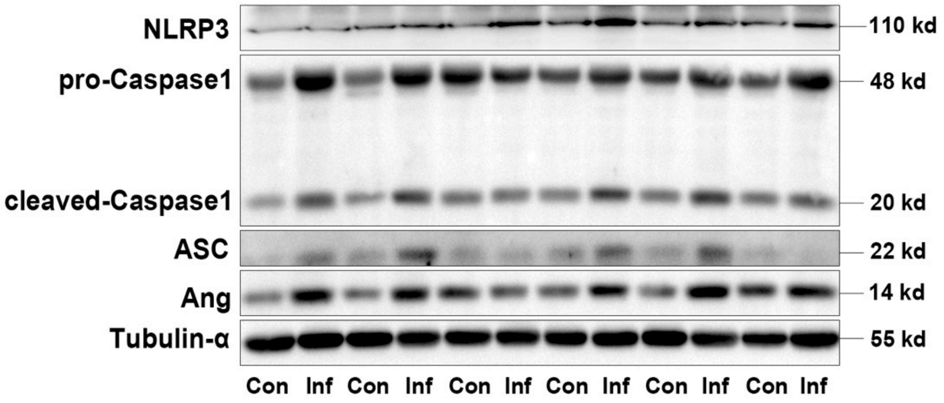

Supplementary Figure S2

Uterine tissues were analyzed by immunoblotting for NLRP3, pro-Caspase1, cleaved-Caspase1, ASC and Ang protein. Tubulin-α was used as a loading control.

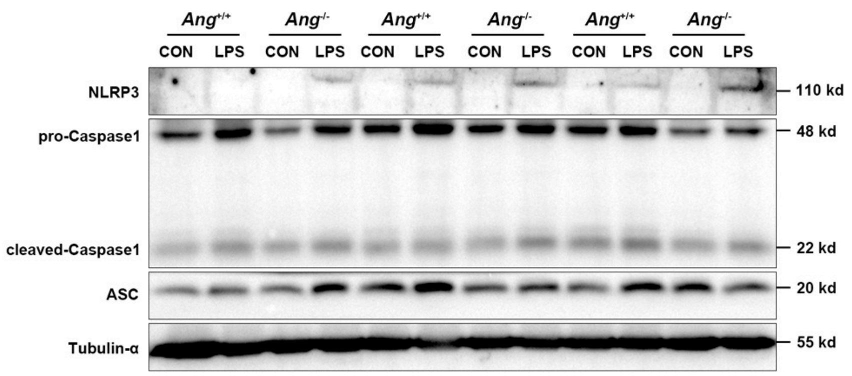

Supplementary Figure S3

Uterine tissues were analyzed by immunoblotting for NLRP3, pro-Caspase1, cleaved-Caspase1 and ASC protein. Tubulin-α was used as a loading control.

Supplementary Table S1. Histopathologic scoring criteria

Feature	Pig	Mice	Score
Hyperemia/Edema	Normal	Normal	0
	Mild	Mild	1
	Moderate	Moderate	2
	Severe	Severe	3
Infiltration with neutrophil	0-1	0-1	0
	2-5	2-5	1
	6-10	6-10	2
	11-20	11-20	3
	20-30	20-30	4
	> 30	30-50	5
		50-100	6
		100-150	7
		>150	8

File S1: Full Western Blot images

Figure 1

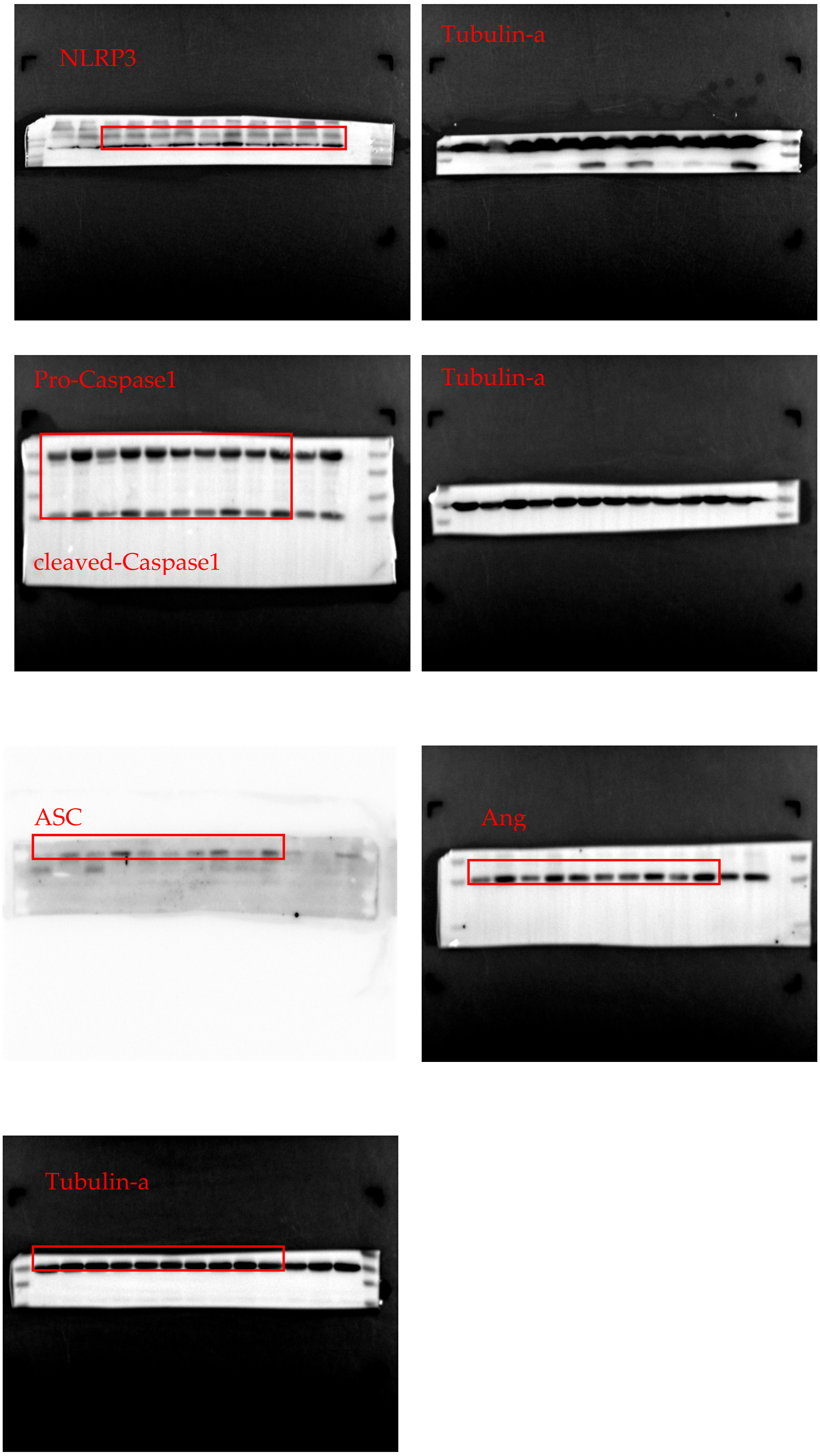

Figure 2

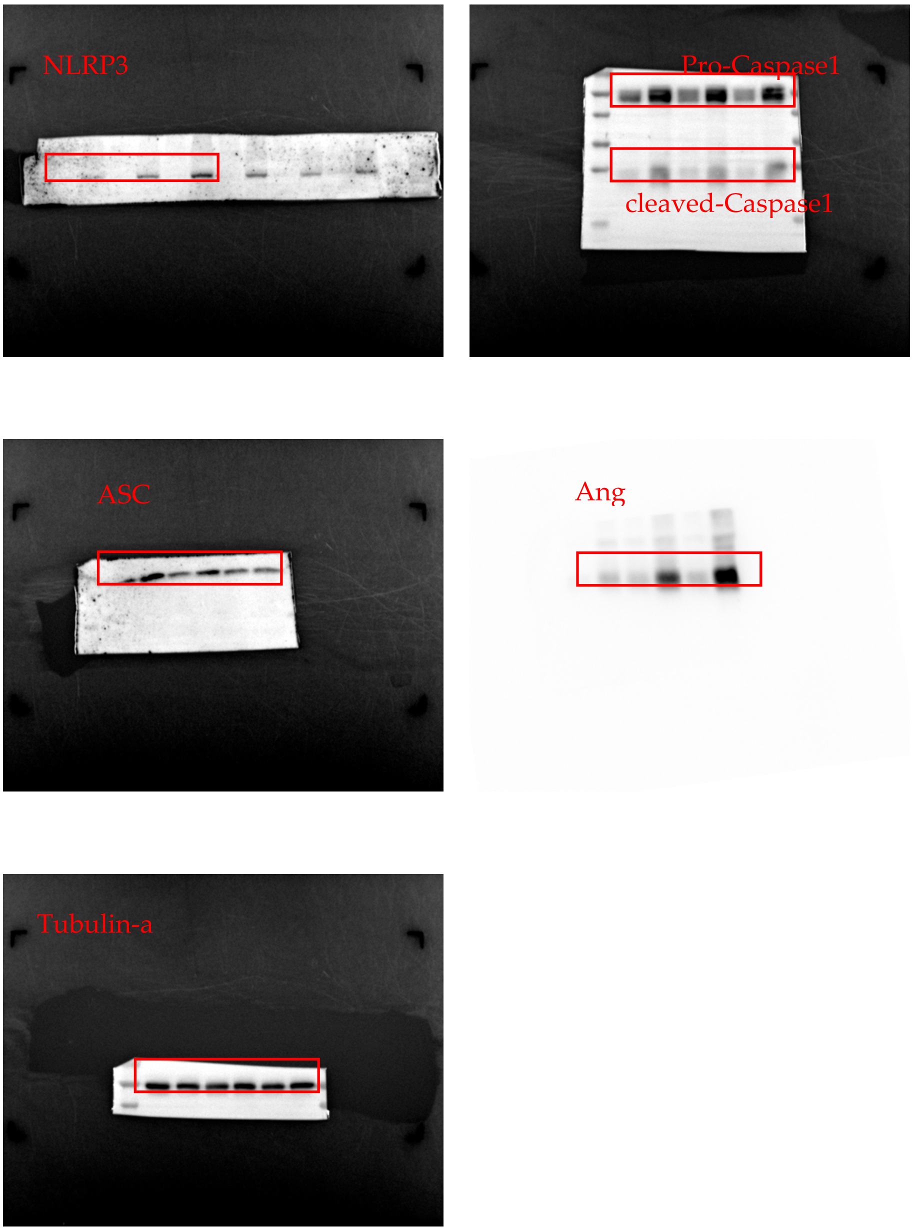

Figure 3

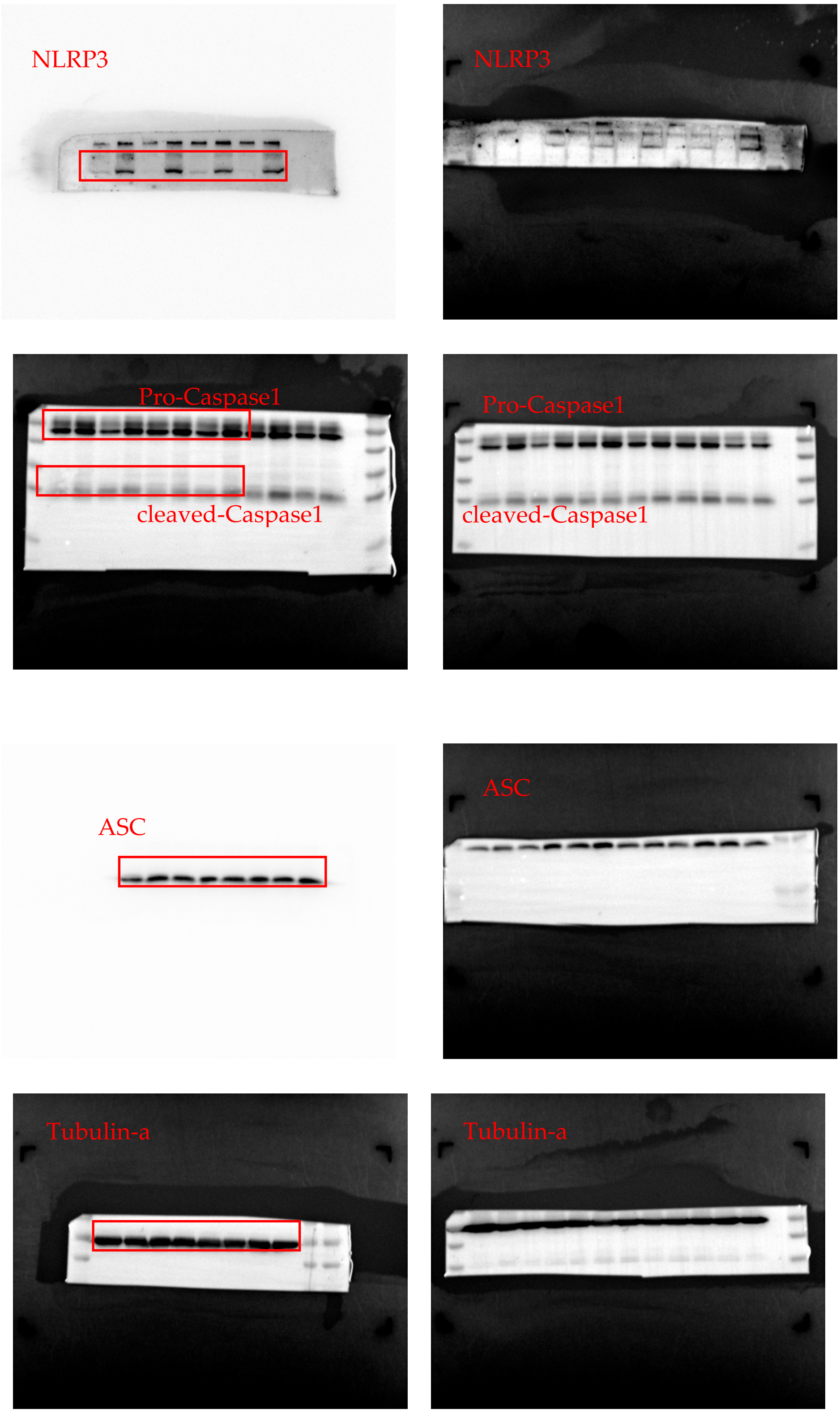

NLRP3
NLRP3
Pro-Caspase1
cleaved-Caspase1
Pro-Caspase1
cleaved-Caspase1
ASC
ASC
Tubulin-a
Tubulin-a

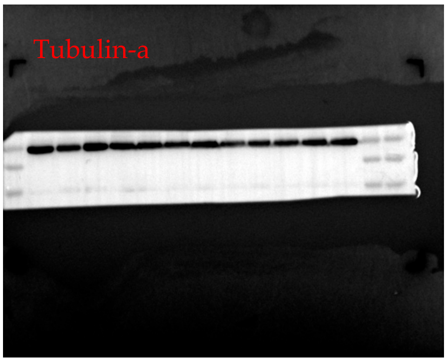
Tubulin-a

Figure 4

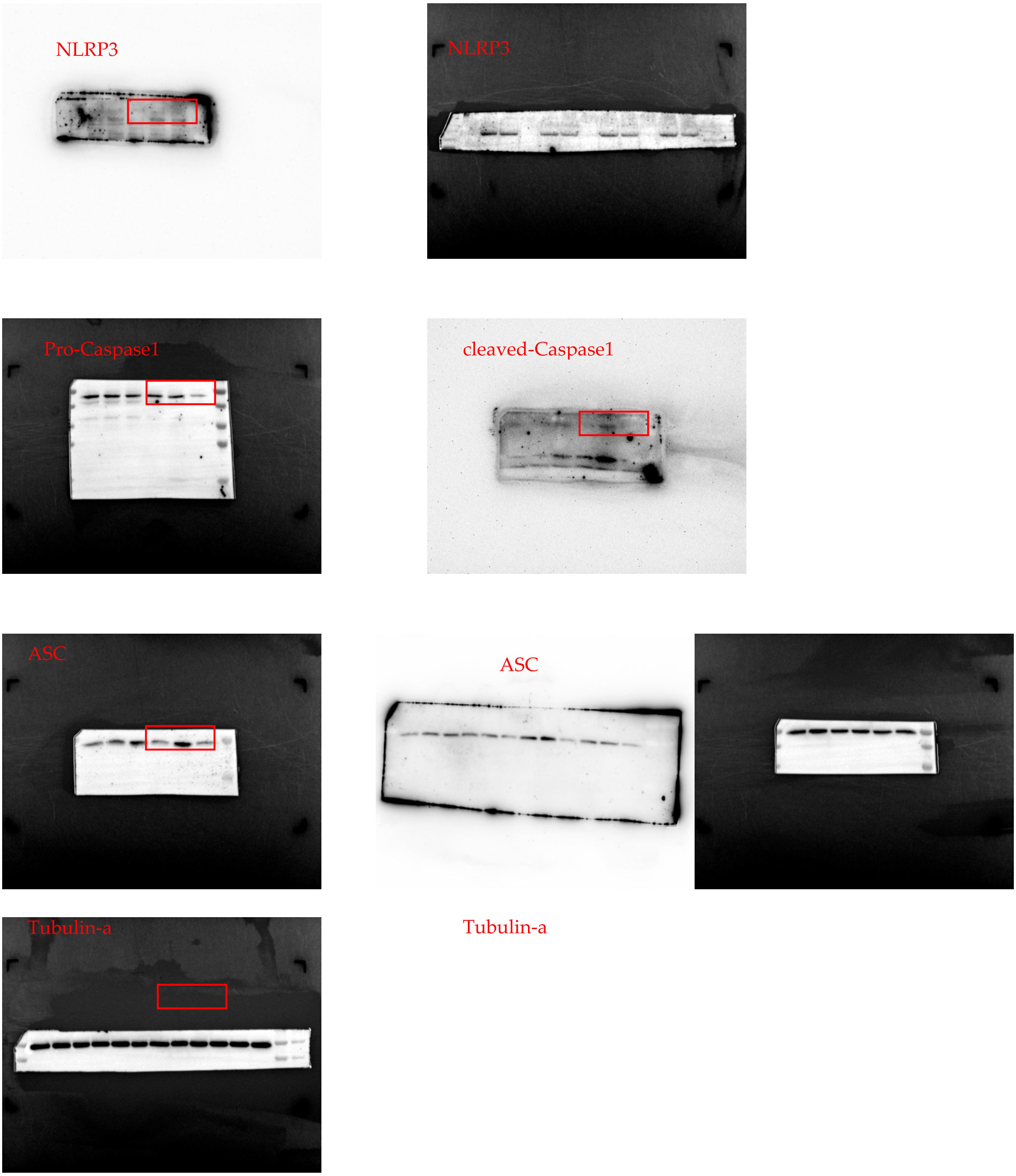
NLRP3
NLRP3
Pro-Caspase1
cleaved-Caspase1
ASC
ASC
Tubulin-a
Tubulin-a
